# Human Adipose Tissue Derived Stem Cells Promote Liver Regeneration in a Rat Model of Toxic Injury

**DOI:** 10.1155/2013/534263

**Published:** 2013-11-07

**Authors:** Eva Koellensperger, Willem Niesen, Jonas Kolbenschlag, Felix Gramley, Guenter Germann, Uwe Leimer

**Affiliations:** ^1^Clinic for Plastic and Reconstructive Surgery, Aesthetic and Preventive Medicine, Heidelberg University Hospital-Ethianum, Voßstraße 6, 69115 Heidelberg, Germany; ^2^Department of Surgery, University of Heidelberg, Im Neuenheimer Feld 110, 69120 Heidelberg, Germany; ^3^Clinic for Plastic Surgery and Severe Burns, BG University Hospital Bergmannsheil, Bürkle-de-la-Camp-Platz 1, 44789 Bochum, Germany; ^4^Department of Cardiology, University of Frankfurt, Theodor-Stern-Kai 7, 60590 Frankfurt, Germany

## Abstract

In the light of the persisting lack of donor organs and the risks of allotransplantations, the possibility of liver regeneration with autologous stem cells from adipose tissue (ADSC) is an intriguing alternative. Using a model of a toxic liver damage in Sprague Dawley rats, generated by repetitive intraperitoneal application of retrorsine and allyl alcohol, the ability of human ADSC to support the restoration of liver function was investigated. A two-thirds hepatectomy was performed, and human ADSC were injected into one remaining liver lobe in group 1 (*n* = 20). Injection of cell culture medium performed in group 2 (*n* = 20) served as control. Cyclosporine was applied to achieve immunotolerance. Blood samples were drawn weekly after surgery to determine liver-correlated blood values. Six and twelve weeks after surgery, animals were sacrificed and histological sections were analyzed. ADSC significantly raised postoperative albumin (*P* < 0.017), total protein (*P* < 0.031), glutamic oxaloacetic transaminase (*P* < 0.001), and lactate dehydrogenase (*P* < 0.04) levels compared to injection of cell culture medium alone. Transplanted cells could be found up to twelve weeks after surgery in histological sections. This study points towards ADSC being a promising alternative to hepatocyte or liver organ transplantation in patients with severe liver failure.

## 1. Introduction

Liver cirrhosis on the basis of a chronic hepatitis B or C, autoimmune hepatitis, chronic alcohol abuse, primary sclerosing cholangitis, and primary biliary cirrhosis are only a few possible reasons for functional liver insufficiency. Irrespective of the cause of a chronic liver damage fibrosis is the ultimate endpoint. The most common cause of chronic liver disease is chronic viral hepatitis which causes inflammation and necrosis followed by deposition of collagens in a portal and periportal fashion [1]. Currently, therapy is limited to liver organ and rarely hepatocyte transplantation. Both are only accessible for a limited number of patients, due to lack of donor organs and difficulties with hepatocyte supply. Human hepatocytes are difficult to maintain in cell culture, do not expand well in vitro, and tend to dedifferentiate in culture [2]. Mesenchymal stem cells (MSC) represent an advantageous cell type for allogenic transplantation because they are immunoprivileged with low major histocompatibility complex (MHC) I and no MHC II expression, therefore reducing the risk of allogenic transplant rejection [3]. MSC from adipose tissue (ADSC) have been shown to differentiate into hepatocyte-like cells with variable success regarding morphology and function, such as urea formation, glycogen synthesis, cytochrome P450 enzyme activity, and expression of hepatocyte-specific gene transcripts [4, 5]. Recently rat ADSC administered through the penile vein demonstrated a promoted hepatic regeneration in a rat model of hepatic ischemia-reperfusion injury and subsequent hepatectomy by significantly raising the mitotic index, the antiproliferating cell nuclear antigen level and the expression of hepatic regeneration associated proteins and genes [6].

To evaluate ADSC impact on liver regeneration in vivo, studies with experimental liver necrosis need to be conducted. Experimental liver necrosis may be induced by different agents, such as allyl alcohol, CCl_4_, ethionine, and galactosamine. Allyl alcohol, however, is the only toxin that causes a predominately periportal damage—a pattern comparable to that seen in viral hepatitis [1]. To optimize this model of chronic liver disease, the proliferation of the remaining hepatocytes has to be inhibited additionally to prevent the restoration of liver function within a few days. Retrorsine is a pyrrolizidine alkaloid which is selectively taken up by the liver and then metabolized into its bioactive form. The result is the alkylation of proteins and DNA. This, in turn, blocks the cell cycle by inhibiting mitosis and thus prevents normal hepatocyte proliferation lasting for months. Furthermore, retrorsine initiates sinusoidal obstruction by the damage to hepatic sinusoidal endothelial cells [7, 8]. Studies have shown that hepatic tissue damage by sole application of allyl alcohol or the combination of allyl alcohol with retrorsine promotes only a mild proliferation of transplanted cells (especially hepatocytes). An additional two-thirds hepatectomy boosts the proliferation of the transplanted cells significantly [9].

In severe or chronic liver damage with the simultaneous inhibition of hepatocyte proliferation intrahepatic stem cells from periportal areas are activated and migrate towards the centre of the hepatic lobule while differentiating into hepatic and biliary cells. Presumably, also bone marrow derived stem cells are involved in hepatic regeneration [10, 11]. For this reason, we hypothesized that stem cells from a different origin could also play a role in the restoration of the liver function. Considering the lack of donor organs and the risks which accompany allotransplantations, such as infections or transplant failure, and the necessary immunosuppression with its negative side effects, supporting liver regeneration with autologous adipose tissue derived stem cells appears to be an interesting and promising alternative. This is especially true as adipose tissue is sufficiently available in most people and tissue sampling does not cause substantial defects.

## 2. Methods

All chemicals, if not otherwise specified, were purchased from Sigma-Aldrich, Munich, Germany.

### 2.1. Isolation and Culture of Adipose Tissue Derived Mesenchymal Stem Cells

All participants provided their written informed consent to participate in this study. This procedure as well as the informed consent form was approved by the ethics committees. This study was conducted under the guidelines and with the approval of the ethical committees of the University of Heidelberg and of the Medical Association of the District Palatinate, Germany. After informed consent, freshly excised subcutaneous adipose tissue of six healthy female adults with a range of age of 34–59 years (median age 39.5 years) and a BMI of 22–33 (median BMI 26.6) undergoing elective plastic surgery, that is abdominoplasty, was used for isolation of mesenchymal stem cells using a procedure modified from Hauner et al. [12]. Briefly, after removing fibrous tissue, the adipose tissue was washed two times in 1% BSA/PBS, minced, and digested enzymatically by collagenase (collagenase CLS; 220 U/mg, Biochrom AG, Berlin, Germany, 1.5 mg/mL, in 1% BSA/Krebs-Ringer-solution) for 45 minutes under constant shaking at 37°C. Mature adipocytes and connective tissue were separated by centrifugation (700 ×g, 7 min., RT). The sedimented cells were resuspended, passed through a 100 *μ*m mesh filter (Neolab, Heidelberg, Germany), and washed twice with 1% BSA/PBS. After erythrocyte lysis (3 minutes, 155 mM ammonium chloride, 10 mM Potassium bicarbonate, and 0.1 mM EDTA) cells were washed again twice and plated at a density of 2 × 10^4^ cells/cm^2^ in expansion medium. After 12 hours the medium was changed to remove nonadherent cells. Expansion medium was replaced every second day.

Cells were cultivated in expansion medium (60% Dulbecco's Modified Eagle's Medium (DMEM)) (Invitrogen, Karlsruhe, Germany), 40% MCDB-201 (Sigma), 1 × insulin transferrin selenium (Becton Dickinson, Heidelberg, Germany), 10^−8^ M dexamethasone, 0.1 mM ascorbic acid-2-phosphate, 2% fetal calf serum (Biochrom, Berlin, Germany), 100 U/mL penicillin (Biochrom), 0.1 mg/mL streptomycin (Biochrom), 10 ng/mL rhEGF (Miltenyi, Bergisch Gladbach, Germany), and 10 ng/mL rhPDGF-BB (CellSystems, St. Katharinen, Germany). Medium was exchanged every second day. Once the cells reached 70% confluence, they were detached with 0.25% trypsin-EDTA (Biochrom, Berlin, Germany) and replated with 3.5 × 10^3^ cells per cm^2^. Cultures were incubated at 37°C with 5% CO_2_.

### 2.2. Adipogenic Differentiation and Oil Red Staining

Cells were seeded in expansion medium at a density of 24.000 cells/cm^2^. After reaching 90% confluence, adipogenesis was induced by the alternated use of basal medium (5% FCS/DMEM) supplemented with IDI-mix (500 *μ*M 3-isobutyl-1-methylxanthine; 1 *μ*M dexamethasone; 1 *μ*M indomethacin) for 2 days followed by basal medium plus 10 *μ*g/mL insulin for 1 day. The induction cycle was repeated 3 times. To confirm the successful adipogenic differentiation, cytoplasmic triglyceride lipid droplets were stained with the Oil Red O staining method as described previously [13].

### 2.3. Osteogenic Differentiation and Alizarin Red Staining

After seeding with a density of 24.000 cells/cm^2^, cells were grown in expansion medium to 90% confluence. Osteogenic induction was initiated by changing the medium to DMEM containing 5% FCS, supplemented with 50 *μ*M L-ascorbate-2-phosphate, 0.1 *μ*M dexamethasone, and 10 mM *β*-glycerophosphate disodium. Calcium deposition was demonstrated histochemically by alizarin red staining as follows: Monolayers of mineralized MSC were washed twice with excess PBS and fixed with prechilled 70% ethanol for 1 hour at −20°C. After a short washing step with H_2_O, the cell layer was incubated with 40 mM alizarin red (pH 4.2) for 1 minute at room temperature. After aspiration of unincorporated dye, cells were washed twice more with excess H_2_O and once with PBS before microscopic analysis.

### 2.4. Flow Cytometry

hADSC expanded to passage four were examined for surface marker expression using flow cytometry. The following monoclonal antibodies (MAbs) conjugated to fluorochromes were used: anti-CD13-APC, anti-CD31-FITC, anti-CD34-FITC, anti-CD44-APC, anti-CD45-FITC, anti-CD49a-PE, anti-CD73-PE, anti-CD90-APC, anti-CD105-FITC, and anti-CD106-APC (all from Becton Dickinson). Isotype antibodies were included for all fluorochromes.

Cells were detached with 0.25% trypsin-EDTA, incubated with directly conjugated MAbs in FACS-buffer (1% FCS, 0.1% NaN_3_ in PBS) for 30 min on ice, washed twice with FACS buffer, and fixed with 1% paraformaldehyde/PBS. Cells were analyzed using a FACSCanto flow cytometry system (Becton Dickinson). Data acquisition and analysis were performed with Diva software (Becton Dickinson).

### 2.5. CM-Dil Labeling

Cells were detached with 0.25% trypsin-EDTA (Biochrom) and centrifuged with 250 g at room temperature. The supernatant was discharged and the pellet resuspended in 60% DMEM low glucose (1 g/L D-glucose) (Invitrogen), 40% MCDB-201, 1 × insulin transferring selenium (Becton Dickinson), 10–8 M dexamethasone, 0.1 mM ascorbic acid-2-phosphate, 100 U/mL penicillin (Biochrom), and 0.1 mg/mL streptomycin (Biochrom) to a final concentration of 1 × 10^6^ cells/mL. 20 *μ*M CM-DiI (Vibrant CM-DiI, number V22888, Molecular Probes) was added and incubated for 5 minutes at 37°C. Cells were then incubated for another 15 minutes under constant slow shaking at 40°C followed by a centrifugation with 250 g at room temperature. The supernatant was discharged and the pellet washed twice with DMEM low glucose (1 g/L D-glucose). Then the cells were filtered through a 40 *μ*M cell strainer (Becton Dickinson, Falcon) and resuspended in 60% DMEM low glucose (1 g/L D-glucose) (Invitrogen), 40% MCDB-201, 1 × insulin transferring selenium (Becton Dickinson), 10–8 M dexamethasone, 0.1 mM ascorbic acid-2-phosphate, 100 U/mL penicillin (Biochrom), and 0.1 mg/mL streptomycin (Biochrom) to a final concentration of 2 × 10^6^ cells/100 *μ*L.

### 2.6. Animals

This study was approved by the Animal Welfare Committee of the Ruprecht Karls University Heidelberg, Germany, and the Provincial Authority of Karlsruhe, Baden-Württemberg in accordance with the National Animal Welfare Act guidelines. All procedures were conducted according to this approval.

Female Sprague Dawley rats weighing 140–200 g were purchased from Charles River Laboratories. They were maintained on an automatic 12-hour light/dark cycle and were fed standard rat chow (Sniff, Germany) and water ad libitum. After one week of acclimatization, blood was drawn through the tail vein to determine standard levels for cholinesterase (CHE), total protein, albumin, glutamic oxaloacetic transaminase (GOT), glutamic pyruvic transaminase (GPT), iron, alkaline phosphatase (AP), and lactate dehydrogenase (LDH). Then all rats received two intraperitoneal injections of retrorsine, separated by an interval of 13 days, each of 30 mg/kg body weight. Retrorsine was dissolved in HCl (pH 2.5) followed by neutralization by NaOH 0.1 N. 30 days after the second retrorsine injection, animals received 10 repeated injections of allyl alcohol every third day, each of 0.31 mmol/kg body weight. Three days after the last injection of allyl alcohol all animals underwent a two-thirds partial hepatectomy. The right and left medial hepatic lobe and the left lateral hepatic lobe were removed; all other lobes stayed in situ. Animals were randomized into two groups. Group 1 received 2 × 10^6^ SC in 200 *μ*L DMEM injected directly into the remaining right lateral hepatic lobe and group 2 received 200 *μ*L DMEM instead and served as control. The injection entrance hole was closed by a short bipolar electric impulse. After thorough rinsing to remove remaining blood clots, an automatic osmotic pump (2ML4, Alzet, Charles River, Sulzfeld, Germany) was implanted into the abdominal cavity for a continuous release of cyclosporine. The abdominal wall was closed in three layers by sutures. Postoperative care was administered with buprenorphine and carprofen every 12 hours and if needed. Blood was drawn through the tail vein every seven days for eight weeks, starting from day three postoperatively until the animals were sacrificed. The levels of CHE, total protein, albumin, GOT, GPT, iron, AP, and LDH were determined. Blood levels before the first retrorsine injection were stated as standard levels. Every 28 days the automatic osmotic pumps were replaced. Six and 12 weeks after surgery, ten animals were sacrificed for histological analysis of stem cell fate.

### 2.7. Histological Analysis

Liver lobes that were resected during two-thirds hepatectomy as well as those that were excised at the end of the follow-up period two, six, 12, and 16 weeks after the injection of ADSC or DMEM were fixed, paraffin-embedded, and processed to 6 *μ*m sections as follows. Fixed specimens were dehydrated in graded ethanol, embedded in paraffin (Histosec, Merck, Darmstadt, Germany), and cut into serial 6 *μ*m sections on a rotary microtome (Mod. RM2255; Leica-Microsystems, Wetzlar, Germany). Paraffin embedded sections were placed into xylene, followed by graded ethanol (2 × 30 sec 100%, 2 × 30 sec 96%, and 2 × 30 sec 70%) and then incubated for 5 min with 5 *μ*g/mL DAPI (Invitrogen) in PBS. Finally the sections were washed three times with PBS and coverslipped with fluorescent mounting medium (Dako, Hamburg, Germany).

Sections were examined with a fluorescence microscope (DM 2500, Leica) and documented with a CCD-camera (DFC 350 Fx, Leica). Overlay images of the different fluorescence channels were generated with the Leica Application Suite 2.7.0, Leica.

### 2.8. Analysis of Blood Parameters

Analysis of blood parameters was performed using a ADVIA 2400 analyzing system (Siemens Healthcare Diagnostics); all chemicals were purchased from Siemens Healthcare Diagnostics. LDH, GOT, GPT, AP, and CHE were determined by measuring the kinetic enzymatic activity. Iron, total protein, and albumin were detected photometrically.

### 2.9. Statistics

Results were analyzed by the Student's *t*-test. A Mann-Whitney Rank Sum test was used when normality tests failed. *P* < 0.05 was considered statistically significant.

## 3. Results

### 3.1. Determination of Stemness

**Figure 1:**
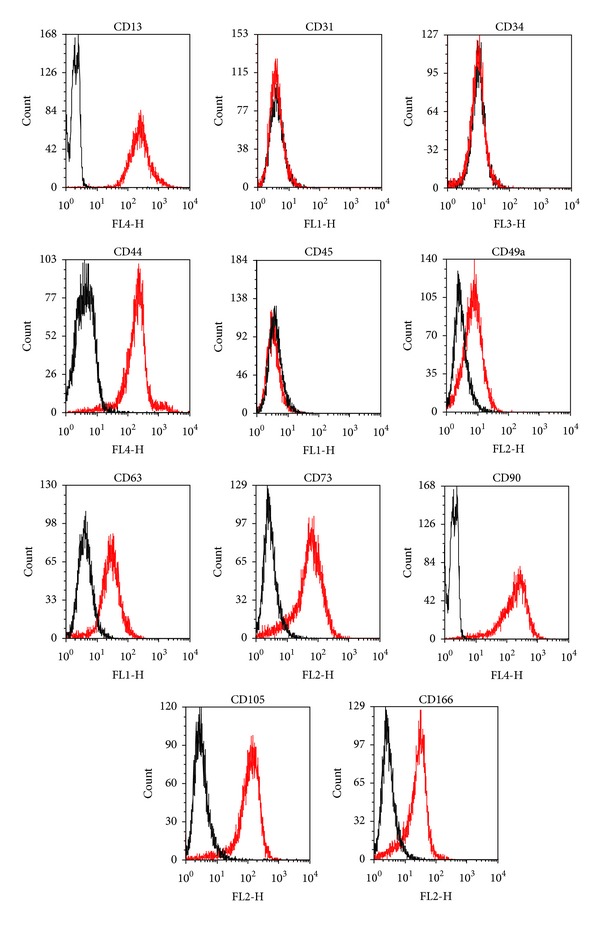
Results of flow cytometry. Black lines represent isotype controls and red lines the analyzed ADSC pool from six different donors. ADSC are positive for CD13, CD49a, CD63, CD73, CD90, CD105, and CD166 and negative for CD31, 34, and 45.

**Figure 2:**
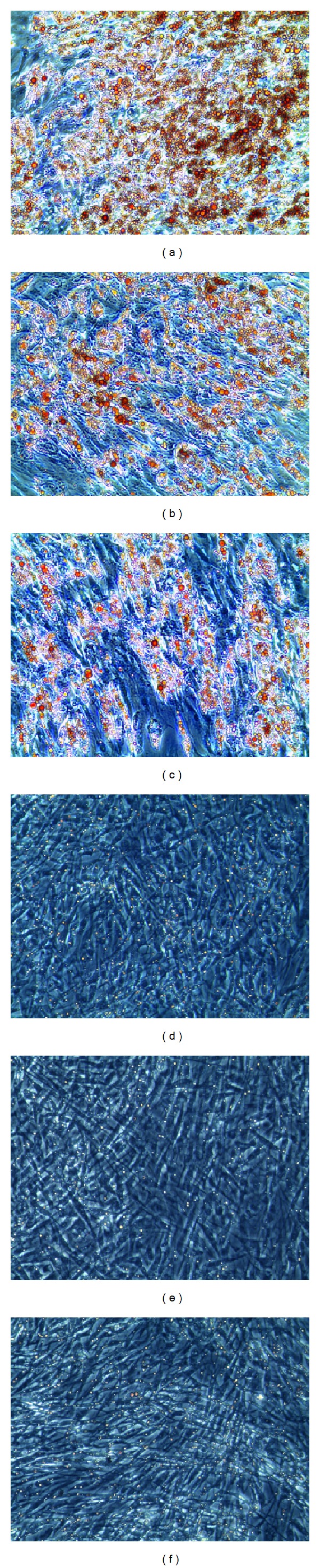
Representative Oil Red O staining of adipogenically differentiated ADSC ((a), (b), and (c)) and not induced control ADSC ((d), (e), and (f)) of three donors demonstrating accumulation of intracellular fat droplets as a sign of adipogenic differentiation in (a), (b), and (c).

**Figure 3:**
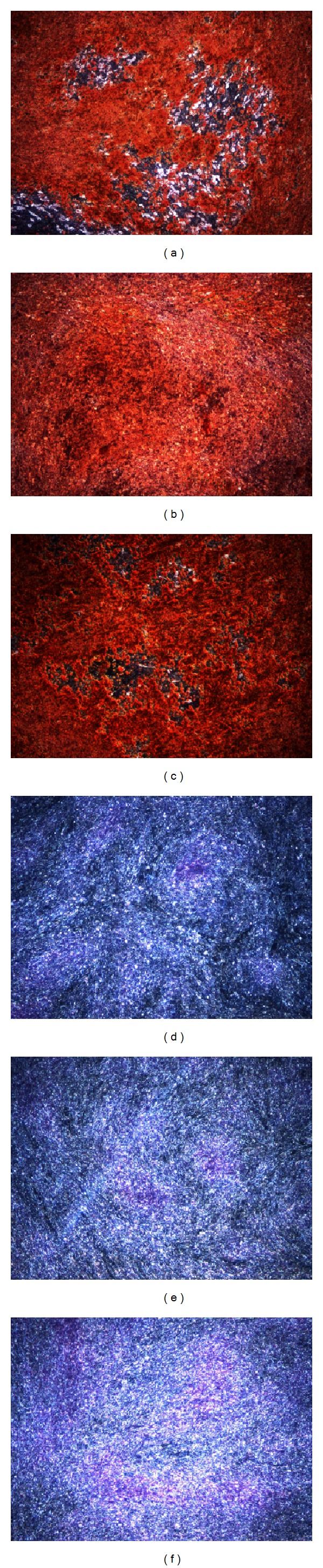
Representative alizarin Red staining in osteogenically differentiated ADSC ((a), (b), and (c)) and not induced control ADSC ((d), (e), and (f)) of three donors demonstrating extracellular calcium deposition as a sign of osteogenic differentiation in (a), (b), and (c).

ADSC were positive for CD13, 44, CD49a, CD63, CD73, CD90, CD105, and CD166. ADSC were negative for CD31, 34, and 45 (Figure 1). The cells therefore meet the minimal consensus criteria for mesenchymal stem cells [14, 15]. Adipogenic and osteogenic differentiation were induced to evaluate multipotent differentiation potential. Adipogenically induced cells showed a significantly higher Oil Red concentration than noninduced control cells in all donors (Figure 2). Osteogenically differentiated ADSC showed high extracellular calcium deposition, analyzed with alizarin red stain. Noninduced control cells did not show extracellular calcium deposition (Figure 3).

### 3.2. ADSC Significantly Raised Albumin, Total Protein Levels, GOT, and LDH

**Figure 4:**
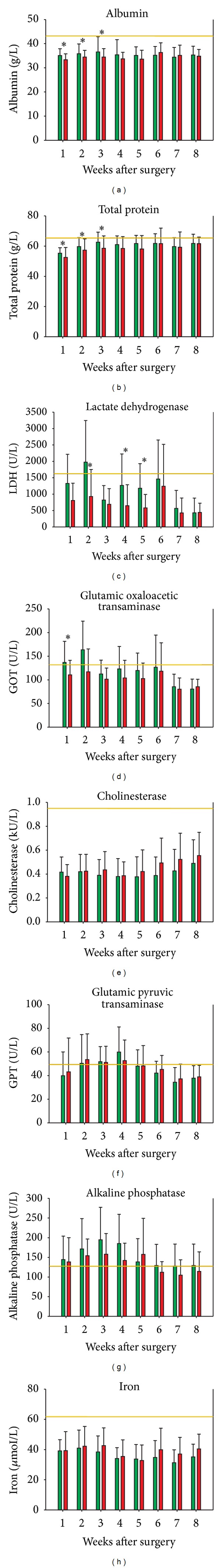
Serum levels of albumin (a), total protein (b), LDH (c), GOT (d), CHE (e), GPT (f), AP (g), and iron (h) in weeks one to eight after surgery. Data are expressed as mean ± standard deviation. Orange lines show normal standard values without toxic liver damage, green bars demonstrate values of cell treated animals, and red bars values of noncell treated control animals. Significant data sets are indicated with asterisks.

Prior to the first application of retrorsine, all blood levels taken from the experimental animals were consistent with published baseline levels for liver enzymes in rats in the literature (Figure 4(a)). They were determined as standard value. Postoperative albumin levels were consistently under the standard value of 43 g/L throughout the entire period of observation. There were statistically significantly higher albumin levels in cell treated animals than in noncell treated control animals in weeks one to three after surgery (*P* = 0.016-0.017). Postoperative total protein levels were higher in cell treated animals than in control animals throughout the whole period of analysis (Figure 4(b)). A return to near standard values (64 g/L) was reached during the third week after surgery in cell treated animals and, with some delay, during the sixth week in control animals. Total protein levels were statistically significantly higher in the cell treated group compared to the control group in weeks two and three after surgery (*P* = 0.015–0.031).

Postoperative LDH levels in control animals lay consistently under the standard value of 1605 U/L (Figure 4(c)). In week eight as an exception LDH levels in cell treated animals were consistently above those in control animals, with a maximum level in week two after surgery. In weeks two, four, and five, LDH was statistically significantly higher in the cell treated group (*P* = 0.003–0.04) compared to the control group.

Postoperative GOT levels in control animals were consistently under the standard value of 130 U/L (Figure 4(d)). GOT in cell treated animals lay higher than in noncell treated animals from the first to the seventh week after surgery, in week one statistically significant (*P* < 0.001). In weeks one and especially two GOT levels in cell treated animals were higher than the standard level. Postoperative CHE-levels were clearly under the standard value of 0.95 kU/L (Figure 4(e)). As a specific marker for a significantly reduced liver function and synthesis, its initial level laid 30 to 50% under the standard value. In both groups CHE levels slowly start to rise six weeks after surgery. A return to standard values was not reached during the eight weeks period of investigation. The nontreated control group had higher CHE levels than the cell treated group; however, there was no statistically significant difference (*P* > 0.05).

Administration of allyl alcohol and retrorsine leads to an elevation of GPT levels before surgery in comparison to the standard level of 48 U/L (Figure 4(f)). Postoperative GPT levels were higher in the control group compared with the cell treated group with a maximum in the second week after surgery. There was no statistically significant difference between cell treated and noncell treated animals.

AP was clearly elevated in comparison to the standard level of 125 U/L after surgery until week six (Figure 4(g)). After week six, alkaline phosphatase levels were higher in cell treated animals compared to control animals. There was no statistically significant difference (*P* > 0.05).

Postoperative iron levels were clearly under the standard level of 61 *μ*mol/L. Apart from week five, animals treated with cells showed lower iron levels than control animals (Figure 4(h)). There was no statistical significantly difference in both groups (*P* > 0.05).

### 3.3. Injected ADSC Could Be Detected up to Twelve Weeks after the Injection in Histological Liver Sections

**Figure 5:**
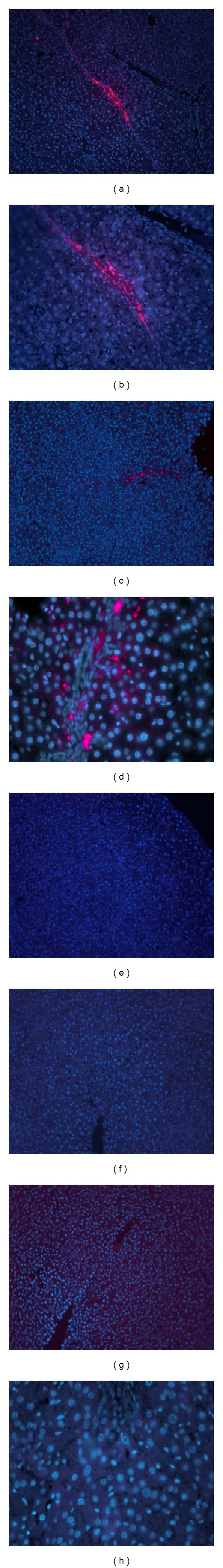
DAPI staining of rat liver lobe sections with CM-Dil-labeled ADSC six weeks after injection ((a), (b)) and twelve weeks after injection ((c), (d)). Control sections from animals six weeks ((e), (f)) and twelve weeks ((g), (h)) after injection of DMEM only, without ADSC. Magnification: (a), (c), (e), (f), and (g) 10x, (b) 20x, and (d) and (h) 40x. Pink signals show CM-Dil labeled ADSC and blue signals show DAPI-stained cell nuclei.

A high variance in the degree of necrosis from rat to rat, lobe to lobe, and section to section could be observed at the time of surgery. Six and twelve weeks after surgery injected liver specimens were paraffin embedded and histological sections were stained with DAPI to visualize the CM-Dil-labeled injected stem cells and cell nuclei (Figure 5). Tumor formation has not been observed in the follow-up period of twelve weeks after the cell treatment.

## 4. Discussion

The aim of this work was to evaluate the possibility of stem cell transplantation in the field of chronic liver diseases, broaden the currently limited therapeutic options, and consequently improve the quality of life and overall survival of affected patients. Therefore, the integration of stem cells into diseased liver tissue and their ability to support liver specific functions were analyzed using a model of a toxic liver damage in Sprague Dawley rats. Allyl alcohol and retrorsine were used to establish a liver damage with a predominantly periportal location, a pattern comparable to that seen in viral hepatitis, the most frequent cause of chronic liver failure [1]. The peak of the Allyl alcohol induced damage occurs approximately 48 hours after injection. A high variance in the degree of necrosis from rat to rat, lobe to lobe, and section to section could be observed in our experiment which is consistent with the results of other authors [16, 17]. This fact makes it very difficult to obtain statistically significant differences due to the inhomogeneity of the individual data. However, our experiments show that the injection of ADSC into a damaged liver after two-thirds hepatectomy might lead to a significantly higher and earlier restoration of liver function compared to the noncell treated control group. This is encouraged by the demonstrated higher albumin and earlier restoration of total protein levels. Negative side effects of cell therapy were not observed, especially no tumor formation, which is consistent with the results of others [18].

Up to date, only few studies have analyzed the possible beneficial effect of human ADSC on liver regeneration. Apart from the fact that these cells are easy to obtain without relevant donor site morbidity, they survive longer in culture with a higher proliferation activity compared to BMSC [19]. The possible role of BMSC in developing liver fibrosis during liver injury, supposably due to interleukin 10 mediation, has been discussed controversially [10, 20–22]. However, we did not see evidence of enhanced liver fibrosis in rats treated with adipose tissue derived stem cells in our experiment.

In contrast to our approach of using undifferentiated ADSC, there have been only few attempts to use hepatocyte-like predifferentiated stem cells for liver regeneration. Banas et al. reported decreasing serum levels of GOT, GPT, and ammonia 24 hours after injecting hepatocytes, differentiated in vitro from human ADSC, into the tail vein of nude mice with liver injury by CCl4 [3]. In addition, Aurich et al. suggested significantly higher engraftment of hepatogenically predifferentiated ADSC compared to undifferentiated ADSC when injected into the spleen of partially hepatectomized mice [4]. However, there are several reasons for the use of undifferentiated stem cells. First of all, undifferentiated MSC are less receptive to oxidative stress than MSC-derived hepatocytes and therefore more likely to survive the initial hypoxic phase after transplantation [23]. Additionally, predifferentiation is more costly and necessitates more handling steps and culture components thus implying a higher risk for contaminations and extra time wasted in cell culture without having the possibility to start urgently needed therapy. Furthermore, undifferentiated BMSC have been shown to differentiate into hepatocyte-like cells without cell fusion when directly administered into the liver of Sprague Dawley rats pretreated with intrahepatic allyl alcohol injections [24]. Most importantly ADSC are thought to induce the vast majority of their in vivo actions by secreting growth factors and cytokines, which then influence various cell functions in diverse surrounding cell types. Thus, a predifferentiation does not only seem to be unnecessary, but also may actually be hindering. In line with that, some recent studies have evaluated the impact of undifferentiated ADSC on hepatic regeneration. Seki et al. have shown that intravenously injected ADSC significantly promoted hepatic regeneration in rats with a two-thirds hepatectomy. At day two after ADSC injection, the gene expressions of IL-6, VEGF, and c-jun were significantly upregulated and different proteins associated with hepatic regeneration and the overall mitotic index were increased. GOT, GPT, and total bilirubin levels, however, were not significantly affected by ADSC injection [25].

Another group of researchers around Seki et al. injected murine ADSC. Their model consisted of an inbred mouse model of diet-induced steatohepatitis in which ADSC were administered through the spleen twice [6]. During a 14-day follow-up period, a decrease in the number of hepatic inflammatory cells, an increase in the albumin expression of hepatic parenchymal cells, and an amelioration of fibrosis could be determined. However, serum parameters were not evaluated and the follow-up period was only limited. In addition, the outcome of mice ADSC cannot directly be equalized with that of human ADSC, since differences between stem cells of varied species are known. Furthermore, to better mimic the diversity of the clinical situation in humans, it might be favorable to use an outbred mouse or rat strain, as in our experiment. In line with that, Harn et al. [26] have used a thioacetamide-induced model of chronic liver damage in outbred Wistar rats. Human ADSC of two different donors were administered directly to the liver with a follow-up period of 28 days. Only at certain points in time were serum albumin and pTT levels significantly higher in ADSC treated animals compared to controls. The injected ADSC were not labeled prior to the administration and, thus, could only be redetected in the liver tissue up to 14 days after the injection by means of immunocytochemistry.

Consistent with these results, the administration of ADSC in our experiment also resulted in a significant increase in albumin levels. Furthermore, we could locate our prelabeled ADSC up to 12 weeks after the injection. In addition, we not only characterized our ADSC by flow cytometry but also further analyzed their proliferation and differentiation capacities, which is still widely considered a prerequisite to determine a cells stemness [14]. Moreover, to display a more general result and to reduce individual differences in the ADSC activity, we decided to use a pool of six different human donors. In contrast to the aforementioned studies, we also performed a surgical reduction of the liver volume and a block of the endogenic hepatocyte proliferation in addition to the toxin-induced liver damage to focus on the regenerative capacity of the ADSC. In consequence, we found not only albumin, as discussed above, to be significantly raised in the ADSC group, but also GOT and LDH.

The significantly increased GOT and LDH levels can be discussed controversially. On the one hand, allyl alcohol also works by the impairment of liver perfusion as a result of the damage to blood vessels and endothelial cells [16]. Liver regeneration also includes restoration of liver perfusion which could lead to an increased outflow of accumulated parameters of cellular damage, such as GOT and LDH, and therefore an increase of the corresponding serum levels as a sign of reperfusion of damaged liver areas. On the other hand, one could also postulate that these heightened serum levels express the ongoing death of cells, possibly also of the transplanted stem cells, and the implemented release of intracellular enzymes such as LDH and GOT. However, injected stem cells can be found from week 1 up to week 12 after surgery in histological sections (Figure 5). No clearing reactions of dead stem cells by macrophages were seen and no CM-Dil could be found in macrophages. Thus, we believe, that the increase of GOT and LDH in our experiment is mainly due to the restoration of blood perfusion in the liver with a consecutively increased clearing and outflow of accumulated parameters of cellular damage.

The increase of AP and GPT within the first four weeks after surgery in both, the cell treated and control groups, can be explained by the well-known changes of bile flow after partial hepatectomy [16–18]. During this process, the remaining hepatocytes are exposed to an increased load of bile components. In combination with the toxic necrosis of liver parenchyma this might lead to elevated blood levels of AP and GPT. However, we did not see a significant influence of ADSC on this phenomenon.

It has been shown that the way of administration of MSCs to the liver does have an impact on their distribution and function in liver parenchyma. A direct intrahepatic injection of stem cells causes a widespread dissemination of cells and a higher number of generated hepatocytes whereas an intraperitoneal injection results in a primarily periportal distribution and lower number of hepatocytes [27]. It has been reported that the synthesis of certain plasma proteins, such as albumin, is higher in periportally localized hepatocytes than in those distributed throughout the rest of the lobule. Albumin synthesis decreases with increasing distance of the hepatocytes to the periportal zone towards the center of the lobule [27–29]. However, in our study we could demonstrate a significant increase in albumin synthesis after intrahepatic injection of ADSC compared to the control group. A certain distribution pattern of injected stem cells within the lobules could not be observed. The injected stem cells could be found up to twelve weeks after injection. In prior experiments, administered stem cells could only be redetected for about four weeks [30].

## 5. Conclusion

This study shows that human ADSC can significantly support liver regeneration and function in a rat model of toxic liver damage and stably integrate into diseased liver tissue. These results point to a possible alternative future therapy for patients with acute or chronic liver disease: the transplantation of autologous ADSC. This vision would offer the possibility to overcome the existing hurdles in current therapies such as the lack of organs for liver transplantation and the existing problems with hepatocyte transfer, as these stem cells can easily and almost ubiquitously be obtained and even expanded prior to therapeutic administration. However, to enable clinicians with standardized and safe protocols in this new field of regenerative stem cell medicine further research is needed.
